# Understanding De Novo Bupropion-Induced Psychosis and Its Management Strategies: A Case Report and Literature Review

**DOI:** 10.7759/cureus.73980

**Published:** 2024-11-19

**Authors:** Moujib Omri, Mohamed Ferhi, Catrin Rauschenbach, Alaa Ibrahim, Mariza Oliveira Galvao, Oliver Hamm

**Affiliations:** 1 Psychiatry and Psychotherapy Department, Klinikum Mutterhaus der Borromäerinnen, Trier, DEU; 2 Psychiatry Department, Hôpital Universitaire Mohamed Taher Maamouri, Nabeul, TUN

**Keywords:** brief psychotic episode, bupropion, bupropion psychosis, drug-induced psychosis, medication-induced psychosis

## Abstract

Psychotic manifestations of iatrogenic origin are common in clinical practice, and it is essential to rule out organic and iatrogenic causes before attributing symptoms to psychiatric disorders. Bupropion, an atypical antidepressant used for treating depression and aiding smoking cessation, has been linked to rare instances of psychosis, especially in patients with risk factors like substance use, older age, or history of head trauma. This report describes the case of a 52-year-old man with recurrent depression who developed a bupropion-induced psychotic episode following an increase in dose to 300 mg/day. He presented with persecutory delusions, although he had no history of psychosis or contributing factors. With the withdrawal of bupropion and the addition of risperidone, the patient's psychotic symptoms resolved within a week. The report takes a look at the recent literature since 2010 and the role of bupropion's dopaminergic effects, its ability to lead to psychosis, and the risk factors associated with this problem. Although there are no guidelines, in some cases antipsychotics such as risperidone have been helpful in reversing bupropion-induced psychosis after discontinuation of the drug.

## Introduction

The occurrence of psychotic events of iatrogenic origin is a common phenomenon in clinical practice. However, before concluding that the psychiatric condition is the cause, it is crucial to exclude any organic or iatrogenic etiology. Bupropion, an atypical antidepressant introduced in the 1960s, is known to rarely induce psychotic symptoms, especially in patients with risk factors such as substance use, advanced age, or prior head injuries [1]. Similarly, other medications, including certain antibiotics, anabolic steroids, and dopamine agonists, have also been associated with psychosis in rare cases [2,3]. Structurally related to amphetamines, bupropion is an effective treatment for depression and is also widely used in smoking cessation [4]. Nevertheless, the occurrence of psychotic symptoms in patients taking bupropion remains a rarely documented phenomenon in medical literature. A literature review by Javelot et al. identified 22 cases between 1985 and 2009 [1]. At therapeutic doses, however, psychosis remains uncommon, with risk factors such as drug use, advanced age, a history of head trauma, or the co-administration of medications metabolized by the cytochrome P450 system, particularly CYP2D6, often being observed [5]. We present here a rare case of a de novo psychotic event in an adult with no history of psychosis, treated with bupropion for severe unipolar depression. These symptoms may appear several weeks after starting treatment. The present case illustrates the clinical features of a bupropion-induced psychotic state, as well as the therapeutic strategy adopted for its management.

## Case presentation

A 52-year-old divorced man and father of two, of middle socioeconomic status, was admitted twice within the last three years to our psychiatry department for recurrent depressive episodes. He had been diagnosed with recurrent depressive disorder and had undergone treatment with a variety of antidepressant medications, including escitalopram, paroxetine, and agomelatine, all of which were prescribed at their maximum therapeutic doses. Despite the patient’s compliance with the prescribed treatment and the initial partial improvement, he experienced a relapse. There was no history of traumatic brain injury, psychotic illness, or drug use. Additionally, there was no history of suicide attempts or recurrent childhood trauma, with the exception of a recent suicide attempt involving an overdose of agomelatine and lorazepam, which required admission to the intensive care unit before transfer to psychiatric care.

Subsequent to this suicide attempt, the patient was transferred from the medical intensive care unit to our department for further management. The initial psychiatric examination indicated the presence of a major depressive episode without psychotic symptoms. The patient exhibited the typical symptoms of depression, including abulia, psychomotor retardation, a depressed mood with affective anesthesia, and instinctual disturbances such as anorexia and sleep-onset insomnia, with a Beck Depression Inventory score of 45, indicating a significant depressive episode. These symptoms were evaluated in accordance with the diagnostic criteria for depression outlined in the Diagnostic and Statistical Manual of Mental Disorders (DSM-5). The patient was initially prescribed quetiapine at an off-label dose of 200 mg/day in conjunction with lorazepam at 2 mg/day, with a plan for gradual reduction. However, due to relapse while the patient was taking selective serotonin reuptake inhibitor (paroxetine) and a lack of improvement in depressive symptoms under agomelatine and quetiapine, treatment with bupropion, reaching a dose of 300 mg/day, was initiated.

His clinical course was marked by the abrupt emergence of a polymorphic delusion of persecution, which lacked clear and coherent organization. The patient was convinced that he was being monitored by the German criminal police and that his movements and phone were under constant surveillance. This delusion was incongruous with the patient's depressive mood, and he exhibited no evidence of thought disorganization, hallucinations, or confusion. Bupropion was initiated at a dosage of 150 mg and increased to 300 mg after one week. The appearance of psychotic symptoms was documented on the day following the increase of the bupropion dosage to 300 mg.

**Figure 1:**
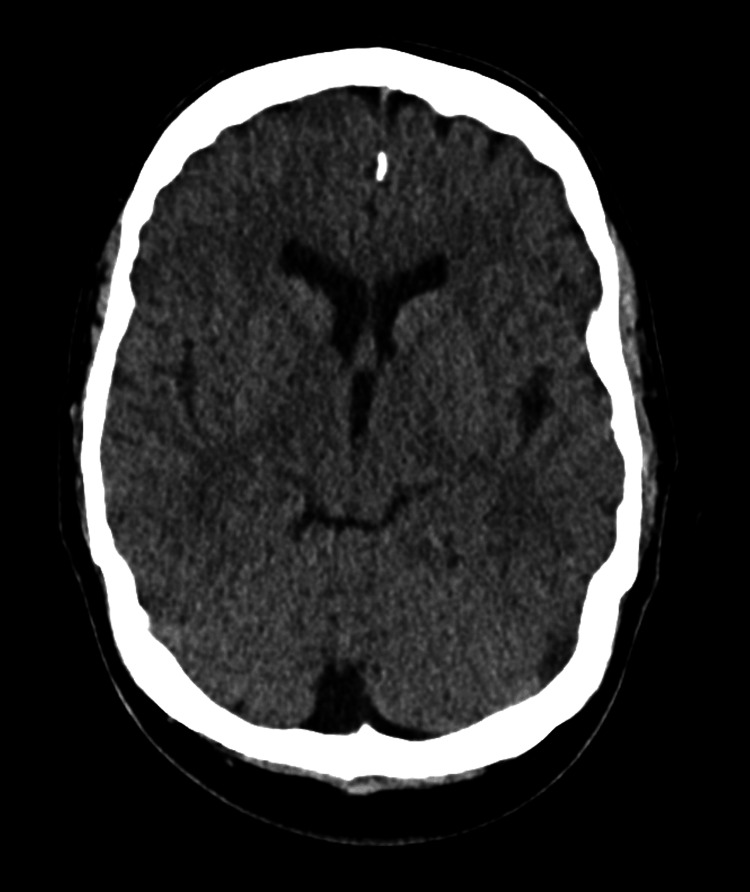
Computed tomography of the head suggesting a normal imaging study.

Following the emergence of psychotic manifestations, the patient underwent a computed tomography (CT) scan of the brain, a series of biological and chemical investigations, and a urine drug test. Laboratory results showed normal complete blood count (CBC), electrolytes, liver function tests, thyroid-stimulating hormone (TSH), urine analysis, and serum C-reactive protein (CRP) levels. With the exception of a positive result for benzodiazepine related to the use of lorazepam, urine drug tests were negative (Table 1). The CT scan of the brain (Figure 1) showed no abnormalities, and the acute psychotic symptoms could not be explained by an organic etiology. The diagnosis of bupropion-induced psychosis was thus established.

**Table 1:** Laboratory test results of the present case.

Tests	Results	Reference
Complete blood count		
Hemoglobin	15.8g/dL	13.8-17.2 g/dL (men), 12.1-15.1 g/dL (women)
Hematocrit	48%	41-50% (men), 36-44% (women)
White blood cells	8.7 x 10³/µL	4.5-11 x 10³/µL
Platelets	242 x 10³/µL	150-450 x 10³/µL
Serum C-reactive protein	3 mg/L	< 5 mg/L
Liver function tests		
Alanine aminotransferase	10 units/L	7-56 units/L
Aspartate aminotransferase	14 units/L	10-40 units/L
Alkaline phosphatase	45 IU/L	40-130 IU/L
Bilirubin, total	0.16 mg/dL	0.1-1.2 mg/dL
Albumin	4.5 g/dL	3.4-5.4 g/dL
Kidney function tests		
Creatinine	0.69 mg/dL	0.6-1.2 mg/dL (men), 0.5-1.1 mg/dL (women)
Estimated glomerular filtration rate	109 mL/min/1.73 m²	>90 mL/min/1.73 m²
Thyroid function tests		
Thyroid-stimulating hormone	0.8 µIU/mL	0.4-4.0 µIU/mL
Free thyroxine	1.1 ng/dL	0.9-1.7 ng/dL
Urine drug testing		
Amphetamin	Negative	Negative
Cannabinoid	Negative	Negative
Cocain-Screening	Negative	Negative
Barbiturate	Negative	Negative
Benzodiazepine	Positive	Negative
Metamphetamine	Negative	Negative
Methylendioxymethamphetamine	Negative	Negative
Phencyclidine	Negative	Negative

Subsequently, bupropion was discontinued abruptly, and risperidone was introduced at a dose of 2 mg/day. This modification resulted in a favorable outcome within one week, with the complete resolution of the delusional symptoms. The patient was then discharged in remission on venlafaxine 150 mg, following the discontinuation of risperidone after a two-week period.

## Discussion

Bupropion is an antidepressant first developed in the United States and subsequently approved by the Food and Drug Administration (FDA) in 1985 for the treatment of depression [1,4]. In terms of structural composition, bupropion bears a resemblance to amphetamines. Previously known as amfebutamone, bupropion demonstrates both antidepressant activity and efficacy in smoking cessation [1]. It exerts its effects through multiple mechanisms at the cerebral level. The antidepressant effect of bupropion can be attributed to its role as a dopamine transporter (DAT) inhibitor in the striatum and a norepinephrine transporter (NET) inhibitor in the locus coeruleus, with no direct serotonergic activity. In contrast to amphetamines, bupropion does not exhibit direct dopaminergic activity. Despite its proven efficacy, the mechanism of bupropion’s action remains only partially understood [6-8]. Additionally, bupropion possesses anticholinergic properties that enhance its effect on smoking cessation. It is a non-competitive antagonist of nicotinic acetylcholine receptors (nAChR), which may explain its efficacy in reducing nicotine dependence [6,8]. Bupropion may be a beneficial treatment option for patients with bipolar depression, as it has a low risk of triggering manic episodes. The potential for bupropion to relieve symptoms of adult attention-deficit hyperactivity disorder (ADHD) has also been proposed [8]. The dopaminergic hypothesis of psychosis suggests that psychotic symptoms arise from dysregulation of dopaminergic activity across diverse brain regions. It further proposes that hyperactivity in the mesolimbic dopamine pathway is the underlying cause of productive psychotic symptoms, including hallucinations and delusions [9]. Bupropion-induced psychosis may be consistent with the dopamine hypothesis of psychosis due to its agonistic effect on dopamine receptors [8]. This hypothesis, which is supported by the inhibitory effect of antipsychotic medications on dopamine D2 receptors, may explain the increased risk of psychotic symptoms with bupropion, which raises dopamine levels between neurons through the inhibition of dopamine reuptake [8].

A review of the literature reveals that bupropion-induced psychotic episodes are relatively uncommon, with only 22 cases reported between 1985 and 2010 [1]. Nevertheless, additional cases have been documented since. Table 2 provides a summary of the cases reported between 2010 and 2024.

**Table 2:** Documented cases of bupropion-induced psychosis between 2010 and 2024 PTSD: post-traumatic stress disorder; MDMA: 3,4-methylenedioxymethamphetamine (also known as "Molly" and "Ecstasy")

Author, Year	Sexe and Age	Indication	Relevant Medical History	Dose/day	Presentation of Psychiatric Symptoms	Management
Pae, 2010 [10]	Female, 28	Depression	None	300 mg	Visual hallucinations	Bupropion stopped
De la Vaga et al., 2010 [11]	Male, 20	Smoking cessation	Depression, cluster B personality disorder, MDMA, cannabis and cocaine abuse	150 mg	Hallucinations, disorganized behavior, and soliloquies	Unclear if bupropion was discontinued; treatment was initiated with antipsychotics
Farooq and Elliott, 2010 [12]	Female, 47	Depression	Depression, PTSD, cocaine abuse	200 mg	Paranoia, visual hallucinations	Bupropion stopped, paliperidone started
Li et al., 2011 [13]	Male, 84	Depression	Dementia, depression, PTSD, chronic kidney disease	300 mg	Visual hallucination, delirium	Bupropion stopped, risperidone replaced with quetiapine
Korkmaz et al., 2012 [14]	Female, 51	Depression	Depression	300 mg	Visual hallucinations	Bupropion stopped, alprazolam and paroxetine started
Kojima et al., 2013 [15]	Male, 81	Fatigue	Multiple sclerosis, PTSD, intermittent auditory hallucinations	300 mg	Visual hallucinations	Bupropion stopped
Munoli et al., 2014 [16]	Male, 62	Smoking cessation	Delusional disorder, depression	300 mg	Agitation, delusions of persecution, and infidelity	Bupropion stopped, haloperidol and lorazepam started
Castelnovo et al., 2015 [17]	Male, 33	Schizophrenia-related depression	Schizophrenia	300 mg	Visual hallucinations	Bupropion stopped, clozapine reduced
Barman et al., 2017 [5]	Male, 48	Depression	Depression, PTSD, traumatic brain injury	400 mg	Paranoid ideations	Quetiapine and trazodone started
Barman et al., 2017 [5]	Male, 51	Depression	Depression, generalized anxiety disorder	450 mg	Delusions	Aripiprazole added, bupropion reduced
Barman et al., 2017 [5]	Male, 67	Depression	Depression, lacunar infarct	150 mg	Paranoid ideations	Bupropion stopped
Yavaş et al., 2019 [18]	Male, 20	unclear	Cannabis abuse	600 mg	Auditory hallucinations, persecutory delusions	Bupropion stopped, paliperidone, vitamin B12 replacement

Among the 12 cases summarized in Table 2, drawn from 10 publications between 2010 and 2024, the average age of patients was seen to be 49.3 years (range, 20-84 years). There have been nine male and three female patients. The primary indication for bupropion was depression in eight cases [5,10,12-14], followed by smoking cessation in two [12,16]. The average dose administered was 312.5 mg, with a range of 150 mg to 600 mg. Psychotic manifestations mainly included visual hallucinations (six cases [10,12-15,17]), followed by auditory hallucinations and agitation [16,18]. With regard to the patients' medical histories, two of the 12 cases involved individuals with pre-existing psychotic or schizophrenic disorders [16,17], seven cases involved patients with depressive histories [5,10,12,14,16], and three cases involved patients with histories of drug use [11,12,18], specifically cocaine or cannabis. Additionally, four patients had preexisting neurological conditions, such as dementia, brain infarction, multiple sclerosis, or previous head trauma [5,13,15]. The majority of patients discontinued bupropion, with the cessation being accompanied by the initiation of antipsychotic or benzodiazepine therapy in eight cases [5,11-14,16,18]. The case presented in this report was of a 52-year-old patient with no history of psychotic disorders or substance use. Both of these factors are recognized as increasing the risk of dopaminergic hyperactivity and psychosis with bupropion [5]. It is noteworthy that, in contrast to the majority of cases where visual hallucinations are common, this patient presented with only persecutory delusions. Following the initiation of bupropion for a depressive relapse, the patient developed a non-mood-congruent delusion, which resolved within one week after discontinuing bupropion and introducing risperidone. The absence of hallucinations and the lack of risk factors such as prior psychosis or drug use distinguish this case from others documented in the literature. Risk factors for bupropion-induced psychosis include prior drug use. In particular, cocaine is likely to dysregulate the reward system, with bupropion increasing dopamine signaling in a reward circuit that has been sensitized by past cocaine use [12]. Furthermore, a history of traumatic brain injury may increase the risk of psychosis due to structural damage and disruptions in brain connectivity [12]. Bupropion is metabolized by the liver via the cytochrome P450 enzyme, specifically CYP2B6. Bupropion and its metabolites are potent inhibitors of the CYP2D6 enzyme, leading to potential drug interactions with selective serotonin reuptake inhibitors (SSRIs), serotonin-norepinephrine reuptake inhibitors (SNRIs), desipramine, valproic acid, and trazodone [19]. This may affect the sensitivity of the dopaminergic system, potentially resulting in the onset of psychotic symptoms [1,18]. Older patients may exhibit heightened sensitivity to bupropion due to the risk of drug and metabolite accumulation in renal impairment, which may result in cognitive impairment and a higher likelihood of psychosis [13]. 

A definitive, linear correlation between dosage and the emergence of psychotic symptoms has yet to be established. However, reports have documented instances of psychosis at doses as low as 200 mg/day [1,5,11,12]. The polymorphic symptomatology of bupropion-induced psychosis includes paranoia, delirium, visual and auditory hallucinations, and even catatonia [1,5,10-18].

Our case illustrates the efficacy of immediate discontinuation of bupropion and initiation of antipsychotic therapy (in this case risperidone), which resulted in the resolution of symptoms within a week. To manage bupropion-induced psychosis, the drug should be discontinued and then followed by temporary antipsychotic support if necessary. Currently, there are no established standard guidelines for the management of bupropion-induced psychosis. Given the structural similarity between bupropion and amphetamines, antipsychotic treatments that have demonstrated efficacy in the treatment of amphetamine-induced psychosis such as haloperidol, risperidone, or olanzapine may be considered following the discontinuation of bupropion. This can be ascribed to their function in blocking and modulating dopaminergic receptors at the mesolimbic level [20]. The limited number of cases presented in existing recent literature impedes our ability to draw definitive conclusions about the optimal approach to managing bupropion-induced psychosis. This represents a significant limitation of our report, given the necessity for longitudinal studies that account for patient vulnerability in order to develop clear and effective guidelines for managing bupropion-induced psychosis. 

## Conclusions

Bupropion is an effective treatment for depressive disorders and smoking cessation, with potential applications in various other psychiatric conditions. The occurrence of paranoid psychotic phenomena caused by bupropion is rare in the literature. These psychotic symptoms may emerge even at therapeutic doses and are more commonly observed in patients with a history of substance abuse, head trauma, or those taking bupropion in combination with medications metabolized by the cytochrome P450 system, particularly cytochrome P450 2D6 (CYP2D6), which bupropion strongly inhibits. From a therapeutic perspective, a reduction in dosage or the discontinuation of bupropion may prove an effective strategy for the management of psychotic symptoms. Additionally, the use of antipsychotic agents may prove beneficial in the management of these symptoms.
